# The Occurrence of Nephrolithiasis in Gout Patients: A Longitudinal Follow-Up Study Using a National Health Screening Cohort

**DOI:** 10.3390/life12050653

**Published:** 2022-04-28

**Authors:** So Young Kim, Dae Myoung Yoo, Ji Hee Kim, Mi Jung Kwon, Joo-Hee Kim, Jung Woo Lee, Woo Jin Bang, Hyo Geun Choi

**Affiliations:** 1 Department of Otorhinolaryngology-Head & Neck Surgery, CHA Bundang Medical Center, CHA University, Seongnam 13496, Korea; sossi81@hanmail.net; 2 Hallym Data Science Laboratory, Hallym University College of Medicine, Anyang 14068, Korea; ydm1285@naver.com; 3 Department of Neurosurgery, Hallym University College of Medicine, Anyang 14068, Korea; kimjihee.ns@gmail.com; 4 Department of Pathology, Hallym University College of Medicine, Anyang 14068, Korea; mulank@hanmail.net; 5 Division of Pulmonary, Allergy and Critical Care Medicine, Department of Medicine, Hallym University College of Medicine, Anyang 14068, Korea; luxjhee@gmail.com; 6 Department of Orthopaedic Surgery, Yonsei University Wonju College of Medicine, Wonju 26493, Korea; berrybearlee@gmail.com; 7 Department of Urology, Hallym University College of Medicine, Anyang 14068, Korea; 8 Department of Otorhinolaryngology-Head & Neck Surgery, Hallym University College of Medicine, Anyang 14068, Korea

**Keywords:** gout, nephrolithiasis, risk factors, cohort studies, epidemiology

## Abstract

The association of gout with nephrolithiasis has been suggested. The current study investigated the risk of nephrolithiasis in patients with gout. The relationship of nephrolithiasis with gout was assessed according to patient characteristics. Individuals in the Korean National Health Insurance Service–Health Screening Cohort were examined. The 17,043 participants with gout were paired with 68,172 comparison participants. The diagnosis of nephrolithiasis was examined in both the gout and control groups. The possible risk of nephrolithiasis in the gout group was analyzed using a stratified Cox proportional hazards model. Subcategory analyses were conducted according to demographic features and comorbidities. The rate of nephrolithiasis was 3.3% (569/17,043) in the gout group and 2.6% (1786/68,172) in the control group. The adjusted hazard ratio (aHR) of gout for nephrolithiasis was 1.23 (95% confidence intervals [95% CI] = 1.12–1.36) in the overall study population. The < 60 years and male groups showed an increased risk of nephrolithiasis related to gout (aHR = 1.26 [1.13–1.42] for the < 60 years group; aHR = 1.27 [1.15–1.41] for the male group). Regarding comorbidities, all subgroups except for the underweight, overweight, total cholesterol ≥ 240 mg/dL, fasting blood glucose ≥ 100 mg/dL, and CCI score 1 and ≥ 2 groups showed a higher risk of nephrolithiasis in gout patients. The gout patients presented an increased occurrence of nephrolithiasis. The middle-aged, male, and healthy populations showed consistently higher HRs of nephrolithiasis related to gout.

## 1. Introduction

Nephrolithiasis is the most common urological disease, and its prevalence has been rising in recent years [1,2]. The occurrence of nephrolithiasis was assessed to be ~10.6% for men and 7.1% for women in the United States [1,2]. Nephrolithiasis is more prevalent in men than women [3]. However, the increasing modifiable risk factors for nephrolithiasis, including obesity and metabolic diseases, have increased the prevalence of nephrolithiasis in women [3]. The formation of nephrolithiasis can be explained by polygenetic causes and related to a wide range of comorbidities [4]. Chronic systemic diseases of obesity, diabetes, and cardiovascular disease have elevated the risk of nephrolithiasis [2,5]. In addition, renal insufficiency and hyperuricemia, which result in acidic urine pH, can increase the possibility of nephrolithiasis, particularly uric acid nephrolithiasis [4,6]. These morbidities of hyperuricemia and renal diseases are also associated with the occurrence of gout [7,8]. 

Gout is known as one of the most common forms of inflammatory arthritis, and its prevalence is estimated to be approximately 1–4% worldwide [7]. Gout is more common in men and older age groups [7]. In addition to impairment of renal excretion of urate in patients with chronic renal diseases, other chronic diseases, including obesity, diabetes, and cardiovascular diseases, increase the risk of gout via activation of inflammation [9,10,11]. Because gout and nephrolithiasis have similar epidemiologic features and risk factors for chronic diseases, it is predicted that the risk of nephrolithiasis can be higher in gout patients. Indeed, previous studies have presented the association of gout with nephrolithiasis [12,13,14,15]. Moreover, because both gout and nephrolithiasis have sex- and age-specific prevalence, the association of gout with nephrolithiasis can be different according to these demographic factors. However, prior studies mainly had a cross-sectional design, which could not determine causality between gout and nephrolithiasis [12,13,14].

We postulated that the presence of gout can increase the possibility of nephrolithiasis. In addition, it was supposed that there may be differences in the association between gout and nephrolithiasis according to patient characteristics, including age, sex, and comorbid conditions. To test these assumptions, a nationwide cohort population was analyzed for the occurrence of nephrolithiasis using gout and control (non-gout) participants. For secondary outcomes, subgroup analyses were conducted according to age, sex, and comorbid conditions.

## 2. Materials and Methods

### 2.1. Study Population

We used the Korean National Health Insurance Service–Health Screening Cohort data, and a thorough explanation for this cohort is presented in prior works [16,17]. This study was permitted by the ethics committee of Hallym University (IRB No.: 2019-10-023) and adhered to the guidelines of the IRB.

### 2.2. Participant Selection

Participants with gout were selected from a total of 514,866 participants with 615,488,428 medical claim codes (*n* = 20,739). The ICD-10 codes were used in Korean throughout the study period. Among other participants, participants who were identified with M10 (Gout) using an ICD-10 code one time were removed (*n* = 10,221). The rest were selected as a comparison group (*n* = 483,096). Among patients with gout, participants were excluded if their first visit with gout was in 2002 to give 1-year washout periods (*n* = 2451), if they did not have a record of blood pressure (*n* = 1), or if they were diagnosed with N20 (Calculus of kidney and ureter) using ICD-10 codes before gout diagnosis (*n* = 1244). In the comparison group, participants were removed if they had no record since 2003 (*n* = 34).

**Figure 1:**
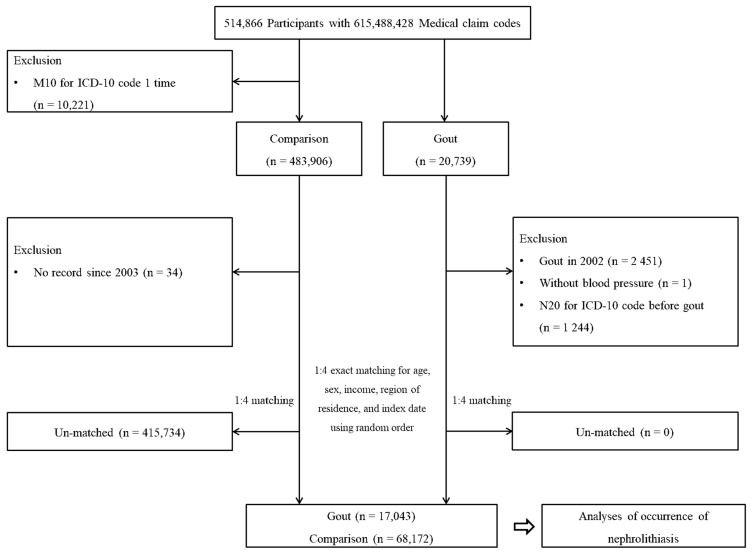
Schematic illustration of the participant selection process used in the present study. Of a total of 514,866 participants, 17,043 gout patients were matched with 68,172 comparison participants for age, sex, income, and region of residence.

Gout patients were paired with comparison participants in a 1:4 ratio for age, sex, income, and region of residence. A random number was allocated to select comparison participants, minimizing selection bias. The index date of each gout participant was defined as the time of their first gout treatment. The index date of comparison participants was defined as the index date of the paired gout patients. Therefore, each matched pair of gout-comparison participants had the same index date. Among the possible candidates for the control participants, we selected only the participants who were not deceased before the index date. During the matching process, 415,734 comparison participants were excluded. Finally, 17,043 gout patients were matched at a ratio of 1:4 with 68,172 comparison participants (Figure 1).

### 2.3. Definition of Gout (Independent Variable)

A diagnosis of gout was identified if participants had gout detected or were treated for gout ≥ 2 times using ICD-10 codes (M10: Gout) [18].

### 2.4. Definition of Nephrolithiasis (Dependent Variable)

Patients who were diagnosed with or treated for nephrolithiasis ≥ 2 times using ICD-10 codes (N20: Calculus of kidney and ureter) were classified as having nephrolithiasis [19].

### 2.5. Covariates

The 10 age groups were defined with 5-year intervals. The 5 income groups were defined (class 1 [poorest]–5 [richest]). Urban and rural areas were grouped [20]. Data on tobacco smoking, alcohol consumption, and obesity were collected [21]. Total cholesterol (mg/dL), systolic blood pressure (SBP, mmHg), diastolic blood pressure (DBP, mmHg), and fasting blood glucose (mg/dL) were measured. The Charlson Comorbidity Index (CCI) was used to reflect comorbidities.

### 2.6. Statistical Analyses

The demographic features of participants with gout were compared with control groups using the standardized difference. Kaplan–Meier analysis and the log-rank test were applied to estimate the cumulative probability of nephrolithiasis in the gout group compared to the comparison group. To analyze the hazard ratios (HRs) with 95% confidence intervals (CIs) for nephrolithiasis in gout patients compared to comparison participants, a stratified Cox proportional hazards model was used. In this analysis, the crude and adjusted models (adjusted for obesity, smoking status, alcohol consumption, total cholesterol, SBP, DBP, fasting blood glucose, and CCI score) were calculated. The analysis was stratified for matched variables.

The study participants were divided according to age (< 60 years old; ≥ 60 years old), sex (males, females), income (low income, high income), and region of residence (urban, rural), and the crude and adjusted models were analyzed using a stratified Cox proportional hazards model. In other subgroup analyses, participants were classified by obesity, smoking, alcohol consumption, total cholesterol, blood pressure, fasting blood glucose, and CCI scores using an unstratified Cox proportional hazards model. The analyses were conducted. A *p* value < 0.05 was considered to be statistically significant. SAS version 9.4 (SAS Institute Inc., Cary, NC, USA) was used for the analyses.

## 3. Results

A total of 3.3% (569/17,043) and 2.6% (1768/68,172) of gout and control participants had nephrolithiasis, respectively (Table 1). The incidence rates of nephrolithiasis were 5.7 per 1000 person-years and 4.4 per 1000 person-years in the gout and comparison groups, respectively. The rates of high levels of total cholesterol, SBP, and DBP and the rates of obesity, alcohol consumption, and high CCI scores were higher in the gout group than in the comparison group.

The HR for nephrolithiasis was 1.29 times higher in the gout group than in the comparison group in the crude model (95% CI = 1.18–1.42, *p* < 0.001, Table 2). In the adjusted model, the gout group had a 1.23 times higher risk of nephrolithiasis than the comparison group (95% CI = 1.12–1.36, *p* < 0.001).

Gout was related to an elevated risk of nephrolithiasis in the < 60 years, male, low-income, high-income, urban, and rural groups (adjusted HR [aHR] = 1.26, 95% CI = 1.13–1.42, *p* < 0.001 for the < 60 years group; aHR = 1.27, 95% CI = 1.15–1.41, *p* < 0.001 for the male group; aHR = 1.32, 95% CI = 1.13–1.55, *p* < 0.001 for the low-income group; aHR = 1.18, 95% CI = 1.04–1.33, *p* = 0.009 for the high-income group; aHR = 1.21, 95% CI = 1.04–1.40, *p* = 0.013 for the urban group; aHR = 1.25, 95% CI = 1.10–1.42, *p* = 0.001 for the rural group). Stratified by comorbid conditions, most subgroups of obesity, smoking status, total cholesterol level, SBP, fasting blood glucose, and CCI scores demonstrated a high risk of nephrolithiasis in gout patients (Table 3). However, the underweight, overweight, total cholesterol ≥ 240 mg/dL, fasting blood glucose ≥ 100 mg/dL, and CCI score 1 and ≥2 subgroups did not show an association of nephrolithiasis with gout.

## 4. Discussion

The presence of gout increases the risk of nephrolithiasis in the population ≥ 40 years of age in Korea. In particular, the middle-aged, male, and healthy (without comorbidities) populations presented an association between gout and the occurrence of nephrolithiasis. This study adds to the knowledge on the risk of nephrolithiasis in gout patients by identifying susceptible populations with gout at risk for the occurrence of nephrolithiasis.

A number of prior studies have suggested a link between gout and the development of nephrolithiasis [12]. A meta-analysis presented a pooled adjusted odds of 1.77 for self-reported lifetime nephrolithiasis in patients with gout (95% CI = 1.43–2.19) [12]. Among gout patients, approximately 39% (95% CI = 31–47) had nephrolithiasis [22]. Moreover, gout patients showed asymptomatic nephrolithiasis rates as high as 24.8%, which were detected only by ultrasonography [23]. However, most prior studies were cross-sectional studies, which were limited to determining the temporality between gout and nephrolithiasis [12].

Several pathophysiologic mechanisms could influence the possibility of nephrolithiasis in patients with gout. Gout has been reported to lower urine pH and increase the risk of uric acid nephrolithiasis [22,24]. In patients with gout, serum uric acid levels and urine pH were correlated with the presence of nephrolithiasis [25]. Acidic urine accelerates the precipitation of uric acid stones with lower amounts of uric acid excretion [24]. In gout patients, a high urinary H+ ion concentration was associated with nephrolithiasis (5.17 ± 3.9 µM/L in patients with nephrolithiasis vs. 3.80 ± 3.01 µM/L in patients without nephrolithiasis) [22].

In addition, inflammatory conditions and related metabolic complications, such as diabetes and metabolic syndrome, in gout patients could increase the risk of nephrolithiasis [26]. Gout has been suggested to be associated with metabolic syndrome by inducing inflammation, adipogenesis, and insulin intolerance [27]. Metabolic syndrome was suggested to increase the risk of nephrolithiasis [28]. A meta-analysis indicated 1.29-fold higher odds of nephrolithiasis in patients with metabolic syndrome (95% CI = 1.11–1.51) [28]. Thus, the components of metabolic dysregulation could mediate the high risk of nephrolithiasis in patients with gout. In addition, molecular biologic pathways promoting inflammatory responses can link gout with nephrolithiasis. For instance, proinflammatory cascades releasing interleukin 1β and activation of NOD-, LRR- and pyrin domain-containing protein 3 (NLRP3) inflammasomes trigger acute flares of gout [29]. NLRP3 was also reported to trigger nephrolithiasis. In an animal study, activation of the NLRP3 inflammasome led to calcium oxalate nephrolithiasis [30].

In subgroup analyses, middle-aged, male, and healthy populations indicated an increased rate of nephrolithiasis in gout patients in the present study. Consistent with this result, a cohort study reported a higher risk of nephrolithiasis related to hyperuricemia in the male population but not in the female population [31]. Healthy young and middle-aged males with hyperuricemia had an approximately 1.72 times higher risk of nephrolithiasis (aHR = 1.44–2.06) [31]. It was suggested that a high blood level of uric acid in menopausal females can attenuate the contribution of gout to the occurrence of nephrolithiasis. Although the exact pathophysiologic mechanisms are still unknown, there are some sex differences in the clinical spectrum and uric acid metabolism [32]. Female gout patients had a later onset of gout, more frequent comorbidities, such as osteoarthritis, hypertension, and renal insufficiency, and higher serum urate levels than male patients [32]. The sex-specific pathophysiology of gout could impose the male-specific association of gout with the possibility of nephrolithiasis. In addition, the middle-aged and healthy population showed a high possibility of nephrolithiasis related to gout in this study. In older or comorbid patients, the association of gout with nephrolithiasis may be mitigated by other mediating risk factors for nephrolithiasis, such as diabetes, hypertension, and renal insufficiencies.

This study analyzed a large cohort population with matched control participants. Covariates included past medical histories, laboratory findings, and lifestyle factors. However, the diagnoses of gout and nephrolithiasis were based on the diagnostic codes chosen by the physician. Because the cohort did not include the results of serum uric acid level tests or imaging examinations for uric acid crystals and nephrolithiasis, the risk of misdiagnosis cannot be excluded. Furthermore, the duration, severity, and management of gout and nephrolithiasis are diverse. Finally, although numerous confounders were adjusted for, there may have been some remaining variables that could impact the occurrence of nephrolithiasis, such as the prescription history of steroids. Future study will be warranted to delineate the risk cumulative comorbidities associated with nephrolithiasis using advanced statistics, such as artificial intelligence.

## 5. Conclusions

The presence of gout increased the possibility of subsequent nephrolithiasis in the adult population. There was a stronger relationship between gout and an increased possibility of nephrolithiasis in gout patients who were middle-aged, male, and had fewer comorbidities. 

## Figures and Tables

**Table 1:** General Characteristics of Participants.

Characteristics	Total Participants		
	Gout (*n* = 17,043)	Comparison (*n* = 68,172)	Standardized Difference
Age (years old, *n*, %)			0.00
40–44	564 (3.3)	2256 (3.3)	
45–49	1951 (11.5)	7804 (11.5)	
50–54	3212 (18.9)	12,848 (18.9)	
55–59	3111 (18.3)	12,444 (18.3)	
60–64	2617 (15.4)	10,468 (15.4)	
65–69	2343 (13.8)	9372 (13.8)	
70–74	1753 (10.3)	7012 (10.3)	
75–79	1033 (6.1)	4132 (6.1)	
80–84	376 (2.2)	1504 (2.2)	
≥85	83 (0.5)	332 (0.5)	
Sex (*n*, %)			0.00
Male	13,567 (79.6)	54,268 (79.6)	
Female	3476 (20.4)	13,904 (20.4)	
Income (*n*, %)			0.00
1 (lowest)	2398 (14.1)	9592 (14.1)	
2	2099 (12.3)	8396 (12.3)	
3	2593 (15.2)	10,372 (15.2)	
4	3602 (21.1)	14,408 (21.1)	
5 (highest)	6351 (37.3)	25,404 (37.3)	
Region of residence (*n*, %)			0.00
Urban	7246 (42.5)	28,984 (42.5)	
Rural	9797 (57.5)	39,188 (57.5)	
Total cholesterol level (mg/dL, mean, SD)	200.2 (40.3)	196.7 (37.5)	0.09
SBP (mmHg, mean, SD)	130.0 (17.3)	127.5 (16.6)	0.14
DBP (mmHg, mean, SD)	80.8 (11.3)	79.3 (10.7)	0.14
Fasting blood glucose level (mg/dL, mean, SD)	101.5 (28.7)	101.9 (30.6)	0.01
Obesity † (*n*, %)			0.27
Underweight	236 (1.4)	1718 (2.5)	
Normal	4352 (25.5)	23,836 (35.0)	
Overweight	4699 (27.6)	19,319 (28.3)	
Obese I	7066 (41.5)	21,670 (31.8)	
Obese II	690 (4.1)	1629 (2.4)	
Smoking status (*n*, %)			0.07
Nonsmoker	9804 (57.5)	38,271 (56.1)	
Past smoker	3263 (19.2)	12,027 (17.6)	
Current smoker	3976 (23.3)	17,874 (26.2)	
Alcohol consumption (*n*, %)			0.10
<1 time a week	8920 (52.3)	39,207 (57.5)	
≥1 time a week	8123 (47.7)	28,965 (42.5)	
CCI score (score, *n*, %)			0.05
0	10,603 (62.2)	46,356 (68.0)	
1	2633 (15.4)	9348 (13.7)	
≥2	3807 (22.3)	12,468 (18.3)	
Nephrolithiasis (*n*, %)	569 (3.3)	1768 (2.6)	0.04

Abbreviations: CCI, Charlson comorbidity index; DBP, diastolic blood pressure; SBP, systolic blood pressure; SD, standard deviation. † Obesity (BMI, body mass index, kg/m^2^) was categorized as < 18.5 (underweight), ≥ 18.5 to < 23 (normal), ≥ 23 to < 25 (overweight), ≥ 25 to < 30 (obese I), and ≥ 30 (obese II).

**Table 2:** Hazard ratio (95% confidence interval) for nephrolithiasis in the gout and control groups with subgroup analyses according to age, sex, income, and region of residence.

Characteristics	No. of Nephrolithiasis/No. of Participants	Follow-Up Duration, Person-Years	Incidence Rate, Per 1000 Person-Years	Hazard Ratios for Nephrolithiasis			
				Crude †	*p*-Value	Adjusted †,‡	*p*-Value
Total participants (*n* = 85,215)							
Gout	569/17,043 (3.3)	99,082	5.7	1.29 (1.18–1.42)	<0.001 *	1.23 (1.12–1.36)	<0.001 *
Comparison	1768/68,172 (2.6)	398,713	4.4	1		1	
Age < 60 years old (*n* = 44,190)							
Gout	387/8838 (4.4)	59,125	6.5	1.33 (1.19–1.49)	<0.001 *	1.26 (1.13–1.42)	<0.001 *
Comparison	1174/35,352 (3.3)	239,037	4.9	1		1	
Age ≥ 60 years old (*n* = 41,025)							
Gout	182/8205 (2.2)	39,957	4.6	1.22 (1.03–1.44)	0.019 *	1.17 (0.99–1.38)	0.075
Comparison	594/32,820 (1.8)	159,676	3.7	1		1	
Males (*n* = 67,835)							
Gout	509/13,567 (3.8)	79,748	6.4	1.35 (1.22–1.49)	<0.001 *	1.27 (1.15–1.41)	<0.001 *
Comparison	1517/54,268 (2.8)	320,749	4.7	1		1	
Females (*n* = 17,380)							
Gout	60/3476 (1.7)	19,334	3.1	0.96 (0.72–1.27)	0.769	0.96 (0.72–1.27)	0.752
Comparison	251/13,904 (1.8)	77,964	3.2	1		1	
Low income (*n* = 35,450)							
Gout	221/7090 (3.1)	39,824	5.5	1.37 (1.18–1.60)	<0.001 *	1.32 (1.13–1.55)	<0.001 *
Comparison	648/28,360 (2.3)	160,604	4.0	1		1	
High income (*n* = 49,765)							
Gout	348/9953 (3.5)	59,258	5.9	1.25 (1.11–1.41)	<0.001 *	1.18 (1.04–1.33)	0.009 *
Comparison	1120/39,812 (2.8)	238,109	4.7	1		1	
Urban (*n* = 36,230)							
Gout	235/7246 (3.2)	42,795	5.5	1.25 (1.08–1.45)	0.003 *	1.21 (1.04–1.40)	0.013 *
Comparison	752/28,984 (2.6)	172,193	4.4	1		1	
Rural (*n* = 48,985)							
Gout	334/9797 (3.4)	56,287	5.9	1.32 (1.17–1.50)	<0.001 *	1.25 (1.10–1.42)	0.001 *
Comparison	1016/39,188 (2.6)	226,520	4.5	1		1	

Abbreviations: CCI, Charlson comorbidity index; DBP, diastolic blood pressure; SBP, systolic blood pressure. * Stratified Cox proportional hazard model, Significance at *p* < 0.05. † Models were stratified by age, sex, income, and region of residence. ‡ Adjusted for obesity, smoking, alcohol consumption, total cholesterol, SBP, DBP, fasting blood glucose, and CCI score.

**Table 3:** Hazard ratio (95% confidence interval) for nephrolithiasis in the gout and comparison groups with subgroup analyses according to obesity, smoking status, alcohol consumption, total cholesterol, blood pressure, fasting blood glucose, and CCI score.

Characteristics	No. of Nephrolithiasis/No. of Participants	Follow-Up Duration, Person-Years	Incidence Rate, Per 1000 Person-Years	Hazard Ratios for Nephrolithiasis			
				Crude	*p*-Value	Adjusted †	*p*-Value
Underweight (*n* = 1954)							
Gout	1/236 (0.4)	1198	0.8	0.45 (0.06–3.37)	0.434	0.44 (0.06–3.40)	0.429
Comparison	16/1718 (0.9)	8821	1.8	1		1	
Normal weight (*n* = 28,188)							
Gout	110/4352 (2.5)	24,466	4.5	1.24 (1.01–1.52)	0.043 *	1.27 (1.03–1.56)	0.026 *
Comparison	499/23,836 (2.1)	138,486	3.6	1		1	
Overweight (*n* = 24,018)							
Gout	121/4699 (2.6)	27,436	4.4	0.95 (0.78–1.15)	0.592	0.95 (0.78–1.15)	0.585
Comparison	530/19,319 (2.7)	114,083	4.6	1		1	
Obese (*n* = 31,055)							
Gout	337/7756 (4.4)	45,982	7.3	1.39 (1.22–1.59)	<0.001 *	1.38 (1.21–1.58)	<0.001 *
Comparison	723/23,299 (3.1)	137,323	5.3	1		1	
Nonsmoker (*n* = 48,075)							
Gout	324/9804 (3.3)	58,807	5.5	1.24 (1.09–1.40)	0.001 *	1.18 (1.04–1.34)	0.009 *
Comparison	1022/38,271 (2.7)	230,020	4.4	1		1	
Past or current smoker (*n* = 37,140)							
Gout	245/7239 (3.4)	40,275	6.1	1.37 (1.19–1.58)	<0.001 *	1.30 (1.13–1.51)	<0.001 *
Comparison	746/29,901 (2.5)	168,693	4.4	1		1	
Alcohol consumption < 1 time a week (*n* = 48,127)							
Gout	320/8920 (3.6)	55,291	5.8	1.26 (1.11–1.43)	<0.001 *	1.22 (1.07–1.38)	0.002 *
Comparison	1130/39,207 (2.9)	247,079	4.6	1		1	
Alcohol consumption ≥ 1 time a week (*n* = 37,088)							
Gout	249/8123 (3.1)	43,791	5.7	1.36 (1.17–1.57)	<0.001 *	1.25 (1.08–1.45)	0.003 *
Comparison	638/28,965 (2.2)	151,634	4.2	1		1	
Total cholesterol < 200 mg/dL (*n* = 46,981)							
Gout	273/8861 (3.1)	49,401	5.5	1.26 (1.10–1.44)	0.001 *	1.22 (1.06–1.40)	0.005 *
Comparison	949/38,120 (2.5)	217,932	4.4	1		1	
Total cholesterol ≥ 200 to < 240 mg/dL (*n* = 27,439)							
Gout	206/5631 (3.7)	33,659	6.1	1.39 (1.19–1.63)	<0.001 *	1.31 (1.11–1.54)	0.001 *
Comparison	574/21,808 (2.6)	130,780	4.4	1		1	
Total cholesterol ≥ 240 mg/dL (*n* = 10,795)							
Gout	90/2551 (3.5)	16,022	5.6	1.15 (0.90–1.47)	0.254	1.11 (0.87–1.42)	0.408
Comparison	245/8244 (3.0)	50,001	4.9	1		1	
SBP < 140 mmHg and DBP < 90 mmHg (*n* = 60,148)							
Gout	345/11,228 (3.1)	61,471	5.6	1.24 (1.10–1.40)	<0.001 *	1.19 (1.06–1.34)	0.005 *
Comparison	1237/48,920 (2.5)	275,056	4.5	1		1	
SBP ≥ 140 mmHg or DBP ≥ 90 mmHg (*n* = 25,067)							
Gout	224/5815 (3.9)	37,611	6.0	1.39 (1.19–1.62)	<0.001 *	1.30 (1.11–1.53)	0.001 *
Comparison	531/19,252 (2.8)	123,657	4.3	1		1	
Fasting blood glucose < 100 mg/dL (*n* = 51,333)							
Gout	341/10,028 (3.4)	60,803	5.6	1.37 (1.21–1.54)	<0.001 *	1.28 (1.13–1.45)	<0.001 *
Comparison	1037/41,305 (2.5)	252,771	4.1	1		1	
Fasting blood glucose ≥ 100 mg/dL (*n* = 33,882)							
Gout	228/7015 (3.3)	38,279	6.0	1.19 (1.02–1.38)	0.024 *	1.15 (0.99–1.34)	0.062
Comparison	731/26,867 (2.7)	145,942	5.0	1		1	
0 CCI score (*n* = 56,959)							
Gout	360/10,603 (3.4)	61,758	5.8	1.37 (1.22–1.54)	<0.001 *	1.31 (1.16–1.48)	<0.001 *
Comparison	1169/46,356 (2.5)	275,735	4.2	1		1	
1 CCI score (*n* = 11,981)							
Gout	96/2633 (3.6)	15,412	6.2	1.25 (0.99–1.58)	0.057	1.17 (0.92–1.48)	0.200
Comparison	271/9348 (2.9)	54,603	5.0	1		1	
≥ 2 CCI score (*n* = 16,275)							
Gout	113/3807 (3.0)	21,912	5.2	1.08 (0.87–1.34)	0.477	1.03 (0.83–1.27)	0.821
Comparison	328 12,468 (2.6)	68,375	4.8	1		1	

Abbreviations: CCI, Charlson comorbidity index; DBP, diastolic blood pressure; SBP, systolic blood pressure. * Un-stratified Cox proportional hazard model, Significance at *p* < 0.05. † Adjusted for age, sex, income, region of residence, obesity, smoking, alcohol consumption, total cholesterol, SBP, DBP, fasting blood glucose, and CCI score.

## Data Availability

Release of the data by the researcher is not legally permitted. All data are available from the database of the Korea Center for Disease Control and Prevention. The Korea Center for Disease Control and Prevention allows data access, at a particular cost, for any researcher who promises to follow the research ethics. The data of this article can be downloaded from the website after agreeing to follow the research ethics.
